# Late Reverse Pupillary Block After Scleral Fixation With Yamane Technique: A Case Report

**DOI:** 10.1155/crop/3923566

**Published:** 2025-12-11

**Authors:** Maria J. Chaves-Samaniego, Gunjan Awatramani, Nazimul Hussain

**Affiliations:** ^1^ Ophthalmology Department, Mediclinic Parkview Hospital, Dubai, UAE; ^2^ Mohammed Bin Rashid University of Medicine and Health Sciences (MBRU), Dubai, UAE

**Keywords:** floppy iris syndrome, lens subluxation, reverse pupillary block, scleral fixation, Yamane

## Abstract

**Introduction:**

The Yamane technique is a transconjunctival, sutureless method for scleral fixation of intraocular lenses (IOL) in cases lacking capsular support. While widely adopted because of its efficacy and safety, rare late complications such as IOL subluxation have been reported. We describe a case of late‐onset reverse pupillary block with recurrent IOL subluxation following Yamane fixation.

**Case Presentation:**

We present a case of a 64‐year‐old male with a history of cataract surgery and ocular hypertension who underwent Yamane scleral IOL fixation in his left eye. Five years later, he presented with cystoid macular edema, which progressed to a full‐thickness macular hole and 360° iris capture of the intraocular lens optic associated with pupillary block. He underwent pars plana vitrectomy, internal limiting membrane peeling, gas tamponade, and IOL repositioning in the sulcus. Despite initial improvement, the patient experienced multiple episodes of IOL subluxation, requiring repeated repositioning and laser iridotomy. Intraoperative findings included IOL tilt and a floppy iris. After the final repositioning, the IOL remained stable at 18‐month follow‐up, with intraocular pressure controlled and visual acuity partially restored.

**Conclusion:**

Although the Yamane technique is generally safe and effective, this case highlights the potential for late reverse pupillary block and recurrent IOL subluxation. Contributing factors included optic–haptic junction stress, iris instability, and improper scleral tunnel architecture. Proper case selection, careful surgical technique, and close follow‐up are essential to minimize the risk of complications.

## 1. Introduction

Scleral fixation of intraocular lenses (IOL) is a surgical technique required in case of damaged, dislocated, or insufficient capsular support, where the implantation of an intraocular lens in the capsular bag or in the ciliary sulcus is not feasible. Some of the leading causes include pseudoexfoliation syndrome, trauma, myopia, or vitreoretinal surgery [1].

The Yamane technique was proposed by Shin Yamane in 2014 and involves transconjunctival sutureless fixation of a three‐piece IOL through two scleral tunnels [2]. After centration of the lens, the haptics are externalized onto the conjunctiva and cauterized to create a flange at each haptic, which is tucked into the scleral tunnels [3]. The flange method involved has become increasingly popular over time because of the increased ease, efficiency, and safety when compared with other methods [4].

However, despite its advantages, this technique is not free of complications. Some reported complications include IOL decentration and tilt, hypotony, uveitis, vitreous hemorrhage, macular edema, and, more rarely, late IOL subluxation or dislocation [5].

The present article reports a case of late‐onset reverse pupillary block with subsequent episodes of recurrent IOL subluxation, occurring 5 years after the primary surgery. It discusses contributing factors, along with a brief review of the literature on potential complications from the Yamane IOL scleral fixation technique, their treatment, and preventive strategies.

## 2. Case Presentation

Sixty‐four y/o male with history of cataract surgery in the right eye 8 years ago and in the left eye 10 years ago. He underwent surgery for lens replacement with Yamane scleral fixation (CT Lucia 602 intraocular lens) in the left eye 5 years later and started treatment for ocular hypertension with bimatoprost 0.01% and brimonidine tartrate–timolol maleate solution 0.2%/0.5% in the same eye a few days after the surgery.

He presented in our hospital for a routine glaucoma checkup. His intraocular pressure (IOP) was 13/10 mmHg in the right and left eyes, respectively, on bimatoprost 0.03% for his left eye. His visual acuity (VA) was 1.0 in the right eye and 0.3 in the left eye. His visual field was within the normal limits in the right eye and showed an advanced double altitudinal scotoma in the left eye (VFI 19%, MD24‐2 ‐25.57 dB, PSD24‐2 7.68 dB). Cystoid macular edema (CME) was detected in the left eye on optical coherence tomography (OCT), so the treatment with bimatoprost 0.03% was replaced by dorzolamide–timolol 0.2%/0.5%. One month later, he developed a full‐thickness macular hole in the left eye and his VA dropped to 0.2.

**Figure 1:**
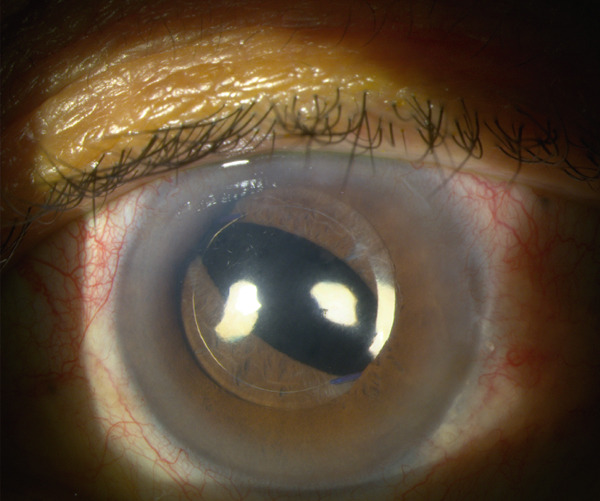
Primary IOL subluxation prior to vitrectomy. CT Lucia 602 hydrophobic acrylic lens with polyvinylidene fluoride (PVDF) monofilament haptics, externalized at 2 o′clock and 8 o′clock positions.

Three days later, he presented with 360° iris capture of the intraocular lens optic associated with pupillary block and IOP 33.2 mmHg (Figure 1). Gonioscopy revealed mild pigmentation of the iridocorneal angle; however, no marked areas of iris atrophy were observed on transillumination. The patient denied any history of similar episodes, ocular trauma, or engagement in high‐impact physical activity. No prior or current use of antiprostatic drugs was reported. Peripheral YAG laser iridotomy (LPI) was performed and his IOP decreased to 15 mmHg under treatment with dorzolamide–timolol 0.2%/0.5% and brimonidine 0.2%.

**Figure 2:**
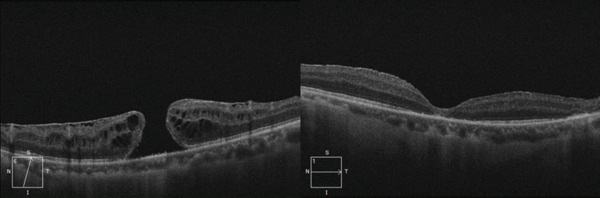
Preoperative and postoperative OCT after pars plana vitrectomy and ILM peeling for full thickness macular hole.

He underwent 25‐gauge pars plana vitrectomy with internal limiting membrane (ILM) peeling, fluid gas exchange with SF6, and lens reposition in the sulcus under general anesthesia (Figure 2). Intracameral acetylcholine was injected in the anterior chamber, and the position of the lens was checked at the end of the surgery. The patient was advised to avoid exercise and Valsalva maneuvers. Postoperative medication included prednisolone, moxifloxacin, and pilocarpine drops.

**Figure 3:**
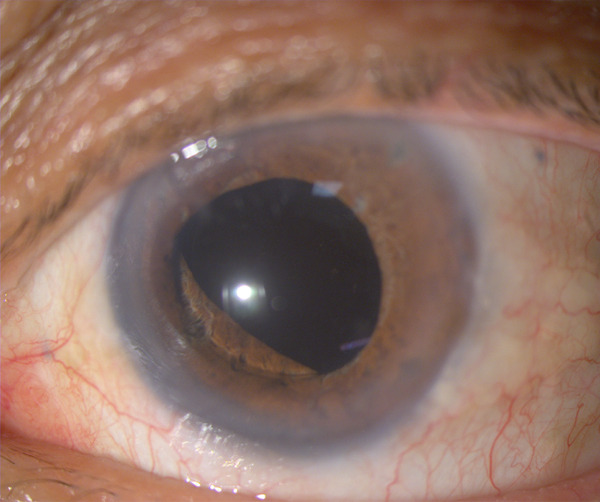
Recurrent partial IOL subluxation with a patent peripheral iridotomy at 1 o′clock position.

His IOP remained at 9 mmHg without hypotensive medication but a diffuse hyphema was noticed from the second day after surgery, resulting in a VA of hand movements. Thirteen days after the surgery, his hyphema was reabsorbed but the patient presented again with a new episode of pupillary block, blocked iridotomies, and new 360 degrees luxation of the lens, with IOP 45 mmHg. This episode was treated as the previous one, reopening his iridotomies with YAG laser, dorzolamide–timolol 0.2%/0.5% and oral acetazolamide. The optic iris capture was resolved by immediately repositioning the optic of the intraocular lens behind the iris under topical anesthesia, and acetylcholine was injected in the anterior chamber. During the intervention, it was found that the lens tended to be tilted with mild anteriorization of the superior optic zone, and the iris was floppy. Moxifloxacin, dexamethasone, dorzolamide–timolol and pilocarpine eye drops were prescribed. His VA improved again in the left eye to 0.2 and his IOP remained at 11 mmHg. Five days after, he had a new episode of partial lens luxation with IOP 16 mmHg (Figure 3). The optic of the intraocular lens was repositioned 30 days later because of patient’s preferences and has remained stable for the past 18 months. OCT showed closed macular hole in the left eye (Figure 2).

In case of new subluxation, this patient was offered some therapeutic options such as pupilloplasty, suturing the subluxated lens to the sclera [6], explanting the IOL [7], or replacing it with a T‐shaped haptics IOL for scleral fixation or an iris clamp fixation IOL [4].

## 3. Discussion

Yamane et al. performed this technique on 100 eyes of 97 patients who had a dislocated/subluxated IOL or aphakia. The best‐corrected VA had improved significantly at 6, 12, and 24 months after surgery. However, at 36 months, there was no statistically significant improvement. Nonetheless, there were postoperative complications noted that included lens capture (8%), vitreous hemorrhage (5%), and CME (1%) [3]. As per the literature review conducted by Shen et al. that compared various techniques for secondary IOL implantation in patients with lack of zonular support, the Yamane technique was found to be one of the most effective for restoring VA with a mean BCVA of 20/36 preoperatively and 20/22 postoperatively [4]. On the other hand, a meta‐analysis conducted by Zhang et al., involving 13 studies did not find any significant difference in the BCVA between the Yamane technique and the scleral suture fixation. Secondly, the time taken during surgery was found to be shorter using the Yamane technique [1].

Reported complications of the scleral Yamane technique can be divided into early and late onset. In a retrospective case‐series reported by Shelke et al., early complications included hypotony (2%), mild vitreous hemorrhage (4%), and raised IOP of > 25 mm Hg (4%). Late complications included pupillary optic capture (2%), retinal detachment (2%), CME (4%), IOL decentration (2%) as well as IOL decentration with tilting (2%) [8].

The most commonly described complications associated with the Yamane technique in the literature include the deformation of the pupil, depigmentation of the iris, CME, and endothelial damage [9]. In a multicenter study conducted in Japan, a post‐op follow‐up period was conducted for over 12 weeks of patients who had undergone the transscleral IOL suture versus the Yamane technique. Thirteen percent of eyes that underwent secondary IOL implantation through the Yamane technique developed CME at 3 months, compared with 1.9% in the conventional technique group [5].

In the study performed by Elsalhy et al., where 33 patients with Marfan’s syndrome had subluxated lenses, the flanged intrascleral haptic fixation technique (Yamane) was utilized. No intraoperative complications were reported along with no follow‐up complications in the 26 out of the 40 eyes. However, IOL decentration was one of the named complications, which was present on the first postoperative day in 12.5% of patients. IOL slippage was found to be present in two cases (5%), which occurred 6 and 11 months after the surgery [10].

Blagum et al. compared the four‐point scleral fixation technique with the Yamane technique in 25 eyes per group. They reported a higher rate of endothelial cell loss (3.5% vs. 0.9%), corneal edema (28% vs. 8%, resolving within 2–3 weeks), increased IOP on day 7 (*p* = 0.025), and a greater incidence of anterior chamber or vitreous hemorrhage (40%, *p* = 0.01) in the Yamane group. One case of IOL displacement requiring repositioning and one case of reverse pupillary block were also noted in the Yamane group [2].

Uveitis‐glaucoma‐hyphema (UGH) syndrome has been described in patients with floppy iris after cataract surgery with lack or insufficient anterior capsular support [6, 7]. UGH syndrome has been reported in 3% of cases of scleral‐fixated IOLs and intermittent pupillary capture of the IOL optic has been reported in 7.9% of these patients [7]. UGH syndrome is an uncommon complication resulting from mechanical irritation caused by a malpositioned intraocular lens, which can traumatically affect the iris, ciliary body, or iridocorneal angle. This may lead to iris transillumination defects, pigment dispersion, microhyphemas, and raised intraocular pressure, and may also be associated with chronic inflammation, secondary iris neovascularization and CME. Although the pathogenesis has not been fully elucidated, proposed mechanisms include activation of innate immune pathways and inflammatory mediator release following mechanical contact between the IOL and ocular tissues, as well as possible contributions from surface‐related reactions or contaminants on the IOL material [11]. It has been described a higher risk in patients undergoing pars plana vitrectomy [12]. Floppy iris seems to be a clear risk factor for optic or haptic capture, UGH syndrome and pupillary block following intrascleral haptic IOL fixation. In patients with notably mobile floppy iris during the surgery, a routine peripheral iridotomy at the time of the IOL fixation should be considered [13].

Pupilloplasty, suturing the subluxated lens to the sclera [6], explanting the IOL [7], or replacing it with a T‐shaped haptics IOL for scleral fixation or an iris clamp fixation IOL [4], are some of the techniques that were considered for this patient.

Lenses with haptics made from polyvinylidene fluoride (PVDF) have demonstrated superior shape memory, resistance to deformation and kinking during their manipulation and melting to create the “mushroom” than those with haptics of polymethylmethacrylate (PMMA) [14]. However, Safran et al. reported some cases of IOL slippage related to PVDF lenses (Zeiss CT Lucia 602) [15].

Some considerations to prevent tilting and IOL slippage during and after the Yamane technique include the following: creating scleral tunnels of similar length, distance, and angle from the limbus, and avoiding excessive manipulation of the haptics and applying strain on the optic–haptic junction during the implantation [15]. The inclusion of a peripheral iridectomy during scleral fixation procedures performed alongside pars plana vitrectomy is associated with a markedly lower rate of reverse pupillary block, improved anterior chamber stability, and a reduced complication profile [16]. Forceps without ridged tips that apply a “platform‐grasping” force are preferable for haptic manipulation [6].

CME is a classical complication of certain intraocular surgeries, resulting from the disruption of the blood ocular barrier. One of the main factors suspected to contribute to the development of CME is secondary irritation of the ciliary body [17], which may lead to anterior rotation of the ciliary body and forward displacement of the lens–iris diaphragm [18]. In this case, the macular edema could be interpreted as a secondary consequence of UGH syndrome, arising from long‐standing mechanical interaction between the intraocular lens and adjacent ocular structures. In addition, a possible association between the macular edema and prior bimatoprost therapy should be considered in the context of the present case [11].

As reported by Schranz et al., reverse pupillary block has been observed in around one‐third of cases involving scleral‐fixated IOL, including those implanted using the Carlevale, Yamane, and Scharioth techniques [19]. Several cases of IOL subluxation have been reported in the literature; however, spontaneous cases (in the absence of trauma or suture breakage) typically occurred in the early postoperative period [2–4, 6–8, 10, 12, 15]. A strength of the present report is that, to our knowledge, it is the first documented case of late‐onset reverse pupillary block with IOL subluxation occurring 5 years after primary surgery using the Yamane scleral fixation technique. One of the main contributing factors in this case appears to be the presence of floppy iris and suspected ciliary body edema. A limitation of this report is that the primary surgery was performed at another institution, and therefore detailed information regarding the surgical technique, lens manipulation, and implantation procedure is unavailable.

Given the frequency of IOL subluxation reported in the literature, the need for lifelong postoperative follow‐up in patients who undergo intraocular surgery must be emphasized. This is particularly important in cases involving scleral fixation techniques, such as the Yamane technique, where long‐term biomechanical factors may predispose to late IOL instability. Continuous monitoring allows early detection and appropriate management of potential complications and optimize long‐term outcomes.

In conclusion, despite the established safety and efficacy of the Yamane scleral fixation technique, this case illustrates the potential for delayed postoperative IOL subluxation. This finding underscores the importance of stringent preoperative assessment, meticulous patient selection, refined surgical execution, and sustained postoperative monitoring to minimize the risk of late complications.

## Ethics Statement

The study adheres to the principles outlined in the Declaration of Helsinki. This case report was approved by the Mediclinic Middle East (MCME) Clinical Research Committee. Written informed consent was obtained from the patient for publication of the medical case and accompanying images.

## Conflicts of Interest

The authors declare no conflicts of interest.

## Funding

No funding was received for this manuscript.

## Data Availability

All data generated or analyzed during this study are included in this article. Further inquiries can be directed to the corresponding author.
